# Molecular Actions of Thyroid Hormone on Breast Cancer Cell Migration and Invasion via Cortactin/N-WASP

**DOI:** 10.3389/fendo.2019.00139

**Published:** 2019-03-07

**Authors:** Ivonne Denise Uzair, Jeremias Conte Grand, Marina Ines Flamini, Angel Matias Sanchez

**Affiliations:** ^1^Laboratory of Signal Transduction and Cell Movement, Institute of Medicine and Experimental Biology of Cuyo, National Scientific and Technical Research Council (CONICET), Mendoza, Argentina; ^2^Tumor Biology Laboratory, Institute of Medicine and Experimental Biology of Cuyo, National Scientific and Technical Research Council (CONICET), Mendoza, Argentina

**Keywords:** triiodothyronine, cortactin, N-WASP, cell motility and invasion, breast cancer

## Abstract

The thyroid hormone triiodothyronine (T3) plays a fundamental role in growth regulation, differentiation, metabolism and cellular movement. These processes are particularly important considering that deregulation of T3 levels could promote abnormal responsiveness of mammary epithelial cells, which may lead to the development and progression of breast cancer (BC). Once cells migrate and invade different tissues, BC metastasis is the main cause of cancer-related death because it is particularly difficult to revert this multistep process. Cell migration integrates several steps that induce changes in cell structure and morphology to promote BC cell invasion. These sequential steps include actin cytoskeleton remodeling, focal adhesion complex formation and, finally, the turnover of branched actin filament networks. In this article, we demonstrate that T3 has the ability to modify the Epithelial-Mesenchymal Transition process. In addition, we show that T3 induces actin cytoskeleton reorganization, triggers focal adhesion formation and, as a consequence, promotes actin nucleation via non-genomic pathway. These events are specifically modulated by T3 via integrin αvβ3 to FAK/paxillin/cortactin/N-WASP/Arp2/3 complex signaling pathway, increasing cell adhesion, migration and invasion of T-47D BC cells. We suggest that T3 influences the progression of tumor metastasis by controlling signaling pathways that converge in cell motility. This knowledge is crucial for the development of novel therapeutic strategies for BC treatment.

## Introduction

Breast cancer (BC) is one of the most common cancer types in women worldwide. Nearly 300.000 new cases of invasive BC are registered every year, and more than 10% of the affected patients die due to this disease (1). Because metastasis is the main cause of death in BC patients, the study of molecular mechanisms that drive cells into an invasive phenotype could offer new perspectives for the development of targeted therapies (2). In order to invade distant tissues, BC cells need to adhere and migrate from the primary tumor, for which actin cytoskeleton reorganization is crucial. Deregulation of the signaling pathways that control actin dynamics marks the onset of cancer progression and metastasis process (3).

Metastasis consists of several steps that include migration of cells away from the primary tumor, their invasion of different tissues and, finally, the generation of a new tumor (4). To achieve these steps, carcinoma cells change their shape, losing their apical-basal polarity to acquire a front-rear polarity that allows cell migration. This process is called Epithelial-Mesenchymal Transition (EMT) (5). This transition involves a downregulation of epithelial proteins expression (E-cadherin) and an upregulation of mesenchymal proteins (vimentin), driving modifications in cell morphology through the reorganization of cytoskeletal architecture (5). Recent studies have demonstrated that the thyroid hormones triiodo-L-thyronine (T3) and tetraiodo-L-thyronine (T4) enhance EMT activity and the metastasis process via integrin αvβ3, reducing E-cadherin and increasing vimentin expression in ovarian cancer cells (6), but the action of T3 on EMT in BC cells remains to be determined.

The thyroid hormones (TH), T3 and T4 regulate cell development, differentiation, metabolism, membrane transport and morphology (7). TH also support BC development by acting as proliferative factors and enhancers of cell migration and invasion (8). T4 has been involved in the promotion of cell proliferation through the ER by a MAPK-dependent pathway in MCF-7 BC cells (9). The effects of TH are generally mediated by genomic mechanisms via nuclear TH receptors that finally regulate specific gene expression. However, in the last decade, several studies have supported the existence of non-genomic or rapid mechanisms triggered by TH, independent of nuclear receptors (10). These rapid actions can be mediated by different plasma membrane-receptors, among them integrin αvβ3 (8). We have recently shown that T3 binds to the integrin αvβ3 receptor, which is highly expressed in several cancer cell lines (8, 10, 11), and it regulates actin cytoskeleton proteins such as Src, FAK, and PI3K, increasing BC cell adhesion and migration (11). Similarly, Lin et al. (12) demonstrated that T3, but not T4, increases Src/PI3K phosphorylation through integrin αvβ3 in human glioma cells. These kinases are over-expressed in different BC cell lines. They activate proteins that are crucial for actinic nucleation, the final step to trigger BC cell motility (13).

One of these proteins is paxillin, a scaffold protein with several key domains for the binding of adhesion molecules and the recruitment of signaling components for actin nucleation (14).

Actinic nucleation, a fundamental mechanism for the branching of actin filaments, is responsible for modifying the cell's cytoskeletal architecture by generating new filaments from pre-existing ones at the cell's leading edge. This crucial step provides the force necessary to trigger cell movement (15, 16). The Arp2/3 complex is one of the main modulators of this process; it is activated by nucleation promoter factors, such as cortactin and N-WASP. Cortactin is able to bind and activate the Arp2/3 complex by its N-terminal region (17). On the other hand, N-WASP belongs to the family of the Wiskott–Aldrich Syndrome Proteins (WASP) and also regulates actinic nucleation by activating the Arp2/3 complex synergistically with cortactin (18). Despite the fact that deregulation of these proteins promotes BC progression, it remains to be studied whether T3 could modulate cell adhesion, migration and invasion, via integrin αvβ3, thus controlling cortactin, N-WASP and Arp2/3 complex activity.

The aim of the present study was therefore to continue elucidating the molecular signal pathway triggered by T3, via integrin αvβ3, on cell morphology and motility in BC cells. In particular, we pretended to deepen our understanding of the rapid actions of T3 on the generation of dynamic structural modifications of the cytoskeleton through key proteins involved in actinic nucleation, representing the starting platform for BC cell migration, invasion, and metastasis.

## Materials and Methods

### Cell Culture and Treatments

The T-47D human breast carcinoma cell line was obtained from the American Type Culture Collection. T-47D cells were grown in RPMI 1640 supplemented with L-glutamine (2 mM), 10% fetal bovine serum (FBS), penicillin and streptomycin under 5% CO_2_ atmosphere at 37°C. Before long treatments, BC cells were kept 24 h in medium containing steroid-deprived FBS. Before experiments investigating non-transcriptional effects, BC were kept in medium containing no FBS for 8 h. T3 was obtained from Sigma-Aldrich, 4-amino-5-(4-chlorophenyl)-7-(t-butyl)-pyrazolo-(3,4-d) pyrimidine (PP2, 10 μM) was from Calbiochem (La Jolla, CA); and Tetraidothyroacetic acid (Tetrac, 10 μM), FAK inhibitor (FAKi, 1 μM), Wiskostatin (10 μM) and CK-666 (4 μM) were from Santa Cruz Biotechnology (Santa Cruz, CA). Whenever an inhibitor was used, the compound was added 45–60 min before starting the active treatments. PP2, FAKi, Wiskostatin and CK-666 were dissolved in DMSO, Tetrac was dissolved in acetone, and Triiodothyronine (T3) was dissolved in RPMI 1640 Medium.

### Immunoblottings

Cell lysates were separated by SDS-PAGE in 8–10% gels and transferred into PVDF membranes. Antibodies used were: p-FAK^Y397^ (611807), FAK (610088) (BD Transduction Laboratories, Lexington, KY); p-FAK (Tyr^397^) (sc-11765-R), cortactin (H-191), p-cortactin (Tyr^466^), paxillin (T-16), p-paxillin (Tyr^118^), E-cadherin (G-10), vimentin (E-5), actin (C-11) (Santa Cruz Biotechnology); α-Tubulin (T9026) (Sigma Aldrich); N-WASP (30D10) (Cell Signaling Technology); p-N-WASP (Ser^484/485^) (Chemicon International); p-Arp2 (Thr237) (Biorbyt). Primary and secondary antibodies were incubated with the membranes using standard techniques. Immunodetection was accomplished using enhanced chemiluminescence and recorded with a quantitative digital imaging system (Chemidoc XRS with Image Lab, Bio-Rad, USA).

### Cell Immunofluorescence

T-47D cells were grown on coverslips. Cells were fixed with 4% paraformaldehyde for 30 min and permeabilized with 0.1% Triton for 5 min. Blocking was performed with PBS containing 3% bovine serum albumin for 30 min at room temperature. Cells were incubated with antibodies against p-paxillin^Y118^; vimentin (E-5) (Santa Cruz Biotechnology); E-cadherin (24E10) (Cell Signaling Technology); p-N-WASP^S484/485^ (Chemicon International); and p-Arp2^T237^ (Biorbyt) overnight at 4 °C, followed by incubation with DyLight^488^/ DyLight^594^ and/or fluorescein-conjugated secondary antibody (FITC 1:150; Vector Laboratories, Burlingame, CA). Cells were then incubated with Texas Red-phalloidin (Sigma-Aldrich, Saint-Louis, MO) for 30 min. After washing, the nuclei were counterstained with or 4'-6-diamidino-2-phenylindole (DAPI) (Sigma-Aldrich, Saint-Louis, MO) and mounted with Vectashield mounting medium (Vector Laboratories, Burlingame, CA). Immunofluorescence was visualized using a Nikon Eclipse E200 microscope and recorded with a high-resolution DP70 Olympus digital camera.

### Gene Silencing With RNA Interference

Synthetic small interfering RNAs targeting paxillin (siRNA paxillin) and control siRNAs were purchased from Santa Cruz Biotechnology (Santa Cruz, CA). The siRNAs were used at the final concentration of 50 nM. T-47D BC cells were treated 48 h after siRNAs transfection. Efficacy of gene silencing was checked with Western analysis and found to be optimal at 48 h.

### Transfection Experiments

Dominant negative constructs for cortactin (*cortactin3YF*, non-phosphorylatable mutant of cortactin) was generously provided by Ph.D John Cooper (Washington University School of Medicine, USA). The inserts were cloned in pcDNA 2AB Flag-cortactin 3YF (19). The plasmids (10 μg) were transfected into T-47D cells using Lipofectamine 2000 (Invitrogen, USA). BC cells were treated 24–48 h after the transfection. Efficacy of transfection was checked with Western analysis and found to be optimal at 36 h.

### Cell Migration Assay

Cell migration was assayed with razor scrape assays. Briefly, a razor blade was pressed through the confluent T-47D BC cell monolayer into the plastic plate to mark the starting line. T-47D cells were swept away on one side of that line. Cells were washed, and 2.0 mL of RPMI 1640 containing steroid-deprived FBS and gelatin (1 mg/mL) were added. Cytosine β-D-arabinofuranoside hydrochloride (Sigma) (10 μM), a selective inhibitor of DNA synthesis that does not inhibit RNA synthesis, was used 1 h before the test substance was added to prevent cell proliferation. Immunofluorescence protocol was performed as previously described in *Immunoflourescence assay*. Only the nuclei were counterstained with or 4'-6-diamidino-2-phenylindole (DAPI) (Sigma-Aldrich, Saint-Louis, MO) and mounted with Vectashield mounting medium (Vector Laboratories, Burlingame, CA). Migration was monitored for 48 h. Cells were digitally imaged and the migration distance was measured by using Nikon Eclipse E200 microscope and recorded with a high-resolution DP70 Olympus digital camera.

### Cell Adhesion Assays

Five hundred thousand cells per well were seeded into 6-well plates on coverslips previously coated with 1% sterile gelatin and exposed to different treatments. The cells were incubated at 37°C for 2 h. Non-adherent T-47D cells were then removed by gently washing with PBS. The attached cells were fixed with 4% formaldehyde and stained with 10% ethanol/crystal violet for 20 min. Cells of attached images were captured and counted in 10 randomly chosen fields per well using a Nikon Eclipse E200 microscope coupled to a high-resolution CCD digital camera, as previously described (20).

### Cell Invasion Assay

Cell invasion was assayed using the BD BioCoatTM Growth Factor Reduced (GFR) MatrigelTM Invasion Chamber (BD Bioscience, USA). In brief, after rehydrating the GFR Matrigel inserts, the test substance was added to the wells. An equal number of Control Inserts (no GFR Matrigel coating) were prepared as control. 0.5 mL of T-47D cell suspension (2.5 × 10^4^ cells/mL) was added to the inside of the inserts. Cytosine β-D-arabinofuranoside hydrochloride (Sigma) (10 μM), a selective inhibitor of DNA synthesis that does not inhibit RNA synthesis, was used 1 h before the test substance was added to prevent cell proliferation. The chambers were incubated for 48 h at 37°C, 5% CO2 atmosphere. After incubation, the non-invading cells were removed from the upper surface of the membrane using cotton-tipped swabs. The cells on the lower surface of the membrane were then stained with Diff-Quick stain. The invading cells were observed and photographed under the microscope at 100 X magnification. Cells were counted in the central field of triplicate membranes.

### Statistical Analysis

All values are expressed as (mean ± SD) of three independent experiments. Statistical analysis of the data was performed using one-way analysis of variance (ANOVA) followed by Barlett's multiple comparisons test using GraphPad Prism 5 software. *P* < 0.05 was considered as statistically significant.

## Results

### T3 Enhances EMT in Breast Cancer Cells

**Figure 1:**
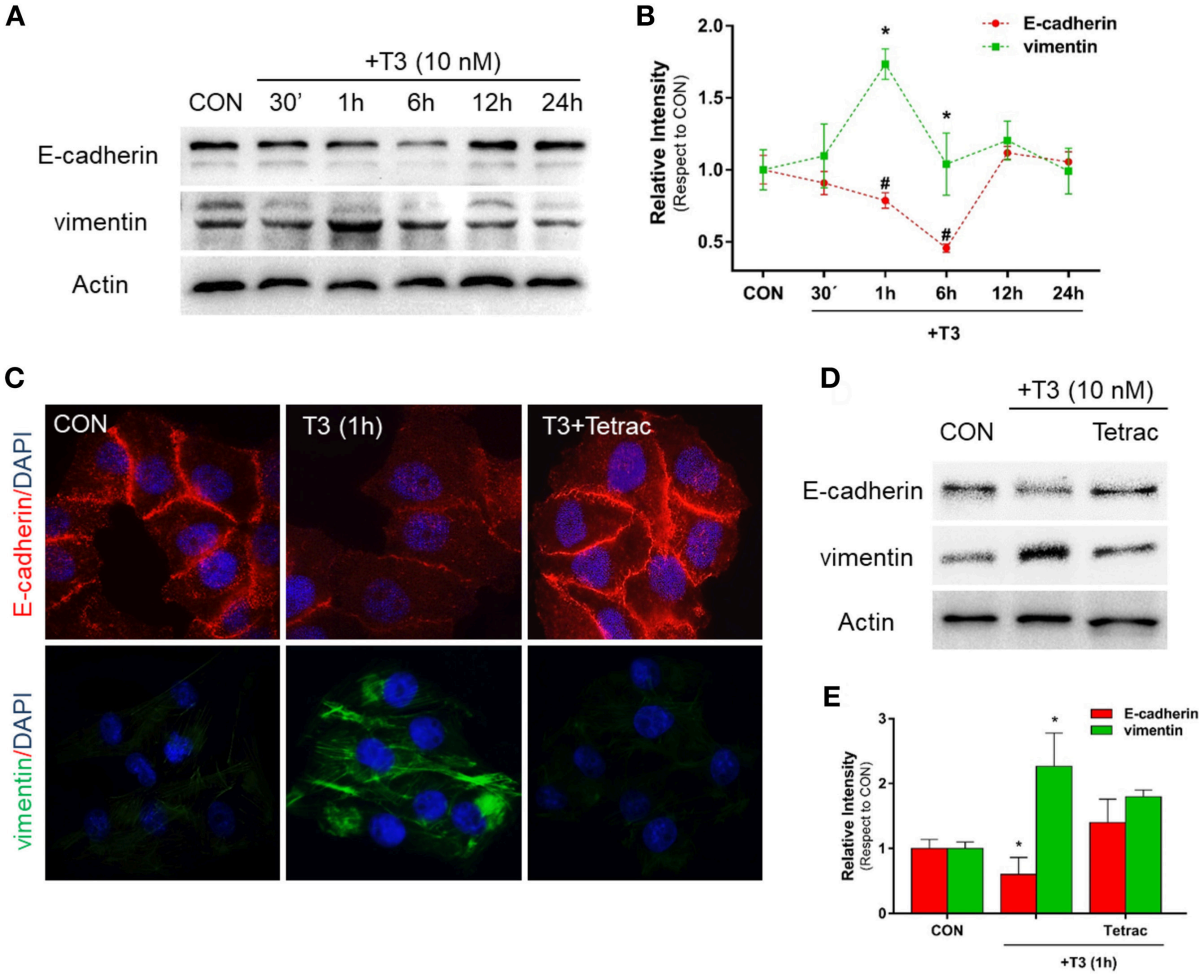
T3 modulates EMT via E-cadherin and vimentin expression**. (A)** T-47D BC cells were treated with T3 for different times (30 min, 1, 6, 12, and 24 h) and Western blot expression patterns for E-cadherin and vimentin were performed. **(B)** E-cadherin and vimentin densitometry values were adjusted to actin intensity, then normalized to the control sample. Results are expressed as mean ± S.D. *P < 0.05 vs. control. **(C,D)** An immunofluorescence assay and Western blot analysis were performed to determine E-cadherin and vimentin expression and localization in BC cells. Cells were treated with T3 for 1 h, in the presence or absence of Tetrac. Cells were stained with E-cadherin linked to DyLight^594^ and vimentin linked to DyLight^488^; nuclei were counterstained with DAPI. CON, Control. **(E)** Each EMT marker densitometry values were adjusted to actin intensity, then normalized to the control sample. Results are expressed as the mean ± S.D. *P < 0.05 vs. control. ^#^*P* < 0.05 vs. control. The experiments were performed in triplicate; representative images are shown.

Epithelial cells have an inherent plasticity that allows them to partially or fully transition into mesenchymal cells by downregulating epithelial and upregulating mesenchymal characteristics in response to an external signal (5). As TH are able to rapidly induce EMT in ovarian cancer cell lines (6), as a first approach we decided to investigate the action of T3 on E-cadherin and vimentin expression, two important markers of epithelial and mesenchymal cells, respectively. After treatment with T3 (10 nM) during different periods (30 min, 1, 6, 12, and 24 h), we observed that T3 induced a progressive decrease in E-cadherin levels starting at 30 min, which became statistically significant at 1 and 6 h and then returned to basal levels at 12 and 24 h (Figures 1A,B). We observed an opposite pattern when we analyzed the action of T3 on vimentin expression. T3 increased vimentin levels starting at 30 min, which became significant at 1 and 6 h and returned to basal levels at 12 and 24 h (Figures 1A,B).

In parallel, we examined the cellular localization of E-cadherin and vimentin with immunofluorescence analysis after 1 h of T3 treatment. In control cells, we observed that E-cadherin was intensely localized in the plasma membrane, whereas vimentin showed a weak cytosplasmatic stain (Figure 1C). After T3 exposure for 1 h, E-cadherin reduced its membrane intensity level whereas vimentin filaments showed an intense cytoplasmatic stain (Figure 1C).

To determine whether T3 initiates its signaling pathway via integrin αvβ3, we treated the BC cells with T3 in the presence of the integrin αvβ3 receptor antagonist tetraiodothyroacetic acid (Tetrac). Tetrac impaired the expression and redistribution of both EMT markers (Figures 1C,D). By western blot analysis we demonstrated that T3 for 1 h induces E-cadherin downregulation and vimentin upregulation, and this effect was impared by Tetrac (Figure 1E), suggesting that T3 promotes EMT activity via integrin αvβ3 in T-47D BC cell.

### Thyroid Hormone T3 Induces Rapid Cytoskeletal and Cell Membrane Remodeling in BC Cells

**Figure 2:**
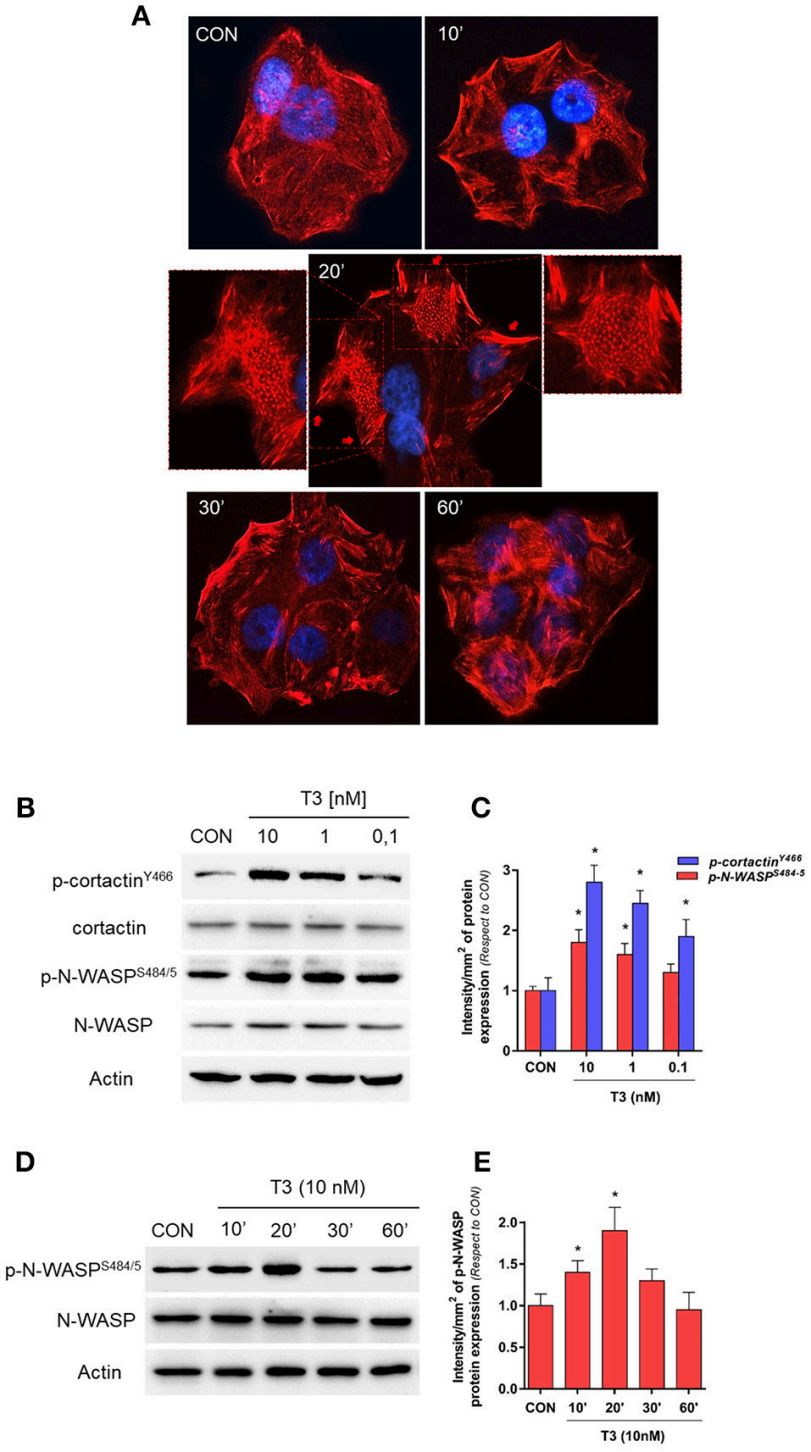
T3 induces dynamic actin remodeling in BC cells. **(A)** T-47D cells were treated for different times with T3 (10 nM). Actin fibers were stained with phalloidin linked to Texas Red (red labeling) and nuclei were counterstained with DAPI (blue labeling). The inserts show the generation of dynamic structural modifications of the cytoskeleton, with the development of protrusive membrane structures, such as lamellipodia and filopodia at higher magnification. **(B,C)** Dose-dependent and **(D,E)** time-dependent cortactin and N-WASP phosphorylation of T-47D cells after T3 treatment. Total cell amount of wild-type cortactin and phosphorylated cortactin (p-cortactin^Y466^) or N-WASP and phosphorylated N-WASP (p-N-WASP^S484/5^) are shown by Western blot analysis. Phospho-cortactin and p-N-WASP densitometry values were adjusted to cortactin and N-WASP intensity, respectively, then normalized to the control sample. *P < 0.05 vs. corresponding control. All experiments were performed in triplicate with consistent results; representative images are shown.

To determine the effects of T3 on BC cell morphology, we analyzed actin cytoskeleton remodeling by means of an immunofluorescence assay. T3 enhanced actin membrane reorganization, which was evidenced by a remodeling of the cytoskeleton toward the plasmatic membrane. The latter led to a thickening of the membrane and, the formation of specialized cell membrane structures involved in the generation of cellular locomotive force, such as lamellipodia, filopodia, and membrane ruffles (Figure 2A, 20 min, red arrows). This event was time-dependent; a maximal remodeling was observed after 20 min of T3 exposure, which returned to basal levels after 60 min (Figure 2A).

Cortactin and N-WASP are two important actin nucleation regulators that are modulated by specific phosphorylation. We evaluated the phosphorylation of cortactin and N-WASP after increasing doses of T3 for 20 min. We observed that the phosphorylation of both proteins was maximal with 10 nM (Figures 2B,C) and therefore used this concentration for the rest of the experiments.

In addition, T3 rapidly induced Ser^484/485^ N-WASP phosphorylation in a time-dependent manner, with a maximal effect at 20 min and a return to basal levels after 60 min (Figures 2D,E).

### T3 Signal to FAK/paxillin Phosphorylation via Integrin αvβ3 Increasing T-47D Cell Migration

**Figure 3:**
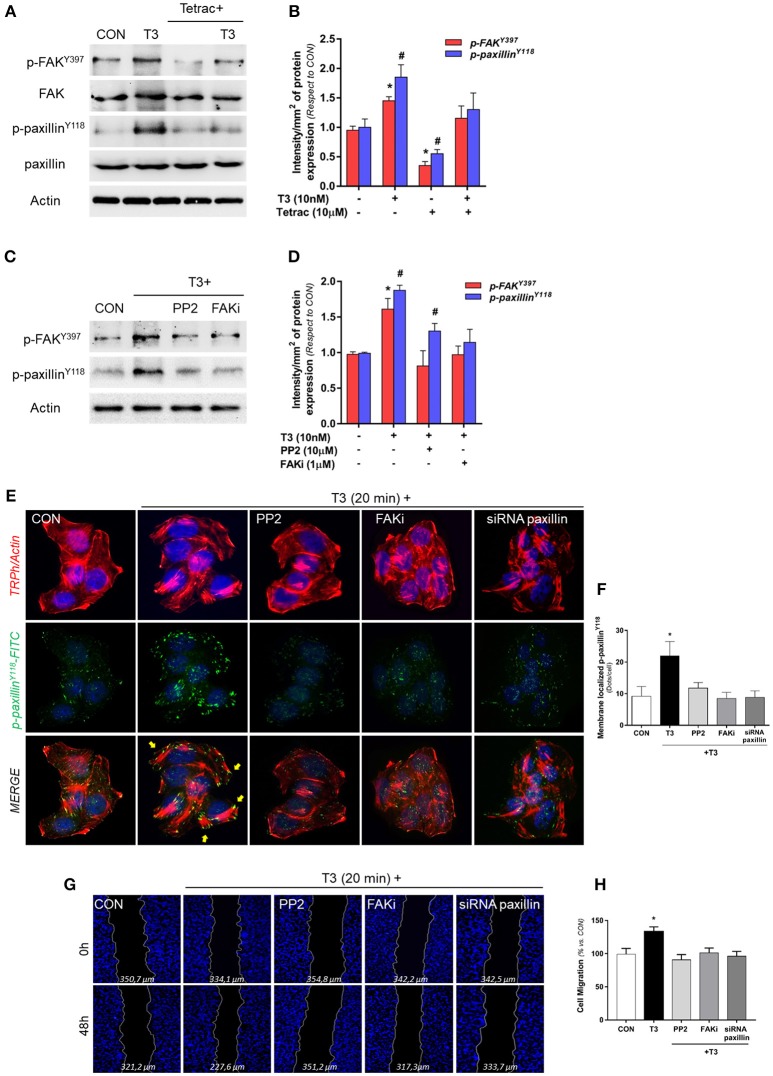
T3 promotes FAK and paxillin phosphorylation through integrin αvβ3**. (A)** T-47D cells were treated with T3 (10 nM) for 20 min in the presence or absence of Tetrac. Total cell amount of wild-type FAK and paxillin, or phospho-FAK and p-paxillin, are shown with Western blot. **(B)** Phospho-FAK and phospho-paxillin densitometry values were adjusted to FAK and paxillin intensity, respectively, then normalized to the control sample. Results are expressed as the mean ± S.D. *P < 0.05 vs. control. ^#^*P* < 0.05 vs. control. **(C)** Cells were exposed to T3 (10 nM) for 20 min in the presence or absence of PP2 (10 μM) and FAK (1 μM), and FAK and paxillin phosphorylation were analyzed through Western blot assay. **(D)** Phospho-FAK and p-paxillin densitometry values were adjusted to FAK and paxillin and/or actin intensity and normalized to the control. Results are expressed as the mean ± S.D. *P < 0.05 vs. control. ^#^*P* < 0.05 vs. control. **(E)** BC cells were stained with anti-phospho-paxillinTyr^118^ linked to FITC, filamentous actin was stained with phalloidin linked to Texas Red and nuclei were counterstained with DAPI. CON, Control. Yellow arrows indicate membrane-localized paxillinTyr^118^-. **(F)** Quantification of the membrane-localized p-paxillin in the different conditions. Results are expressed as Dots/cells (mean ± SD). *P < 0.05 vs. control. Membrane-localized p-paxillin was counted in 40 different cells. The experiments were repeated three times with consistent results. **(G)** Cells were treated with T3 (10 nM) for 48 h in the presence or absence of PP2, FAKi and siRNA paxillin. Representative images are shown. Migration assay was monitored at 48 h by taking photographs. DAPI was used to stain nucleus and Gap closure was quantified with the use of NIH image J software. *P < 0.05 vs. control. **(H)** Cell migration distances were measured; values are presented as % of control. *P < 0.05 vs. control. The experiments were performed in triplicate with consistent results; representative images are shown.

We evaluated the role of diverse protein kinases in the cascades involved in the signaling to cortactin and N-WASP in BC cells. We had previously determined that T3 induces rapid phosphorylation of Src, FAK and PI3K, via integrin αvβ3, and promotes BC cell motility (11). We thus analyzed two central proteins involved in focal adhesion complex formation, FAK and paxillin phosphorylation/activation, after T3 stimulation in T-47D cells. We observed that a rapid pulse of 10 nM T3 (20 min) increased FAK^Tyr397^ and paxillin^Tyr118^ phosphorylation and that this effect was prevented by the integrin αvβ3 receptor antagonist Tetrac (Figures 3A,B). Src and FAK are the main kinases involved in focal adhesion signaling; they are responsible for the recruitment and phosphorylation of paxillin. We therefore used pharmacological inhibitors of Src (PP2) and FAK (FAKi) in cells exposed to T3 and observed that the increase of phospho-FAK^Tyr397^ and phospho-paxillin^Tyr118^ was impaired by PP2 and FAKi, suggesting that T3 induces paxillin phosphorylation via Src and FAK (Figures 3C,D).

To determine the role of paxillin in the formation of focal adhesion complex, we next examined its subcellular localization in the presence of T3 by means of immunofluorescence. Breast cancer cells treated with T3 triggered a significant increase of phospho-paxillin^Tyr118^ at the cell membrane periphery where cortical actin complexes were formed, and this was impaired by the blockade of Src (PP2), FAK (FAKi), and paxillin (siRNAs) (Figures 3E,F).

Finally, we evaluated BC cell migration through a wound-healing assay during exposure to T3 during 48 h. We observed that T3 significantly enhanced BC cell migration; this effect was prevented by the use of PP2, FAKi and the silencing of paxillin with specific siRNAs (Figures 3G,H). Altogether, these results suggest that T3 signals to paxillin through a Src/FAK cascade. When paxillin is phosphorylated, it consequently translocates to the cell periphery, promoting BC cell migration.

### Intracellular Events Linking Activation of Paxillin to Cortactin, N-WASP and Arp2/3 Complex

**Figure 4:**
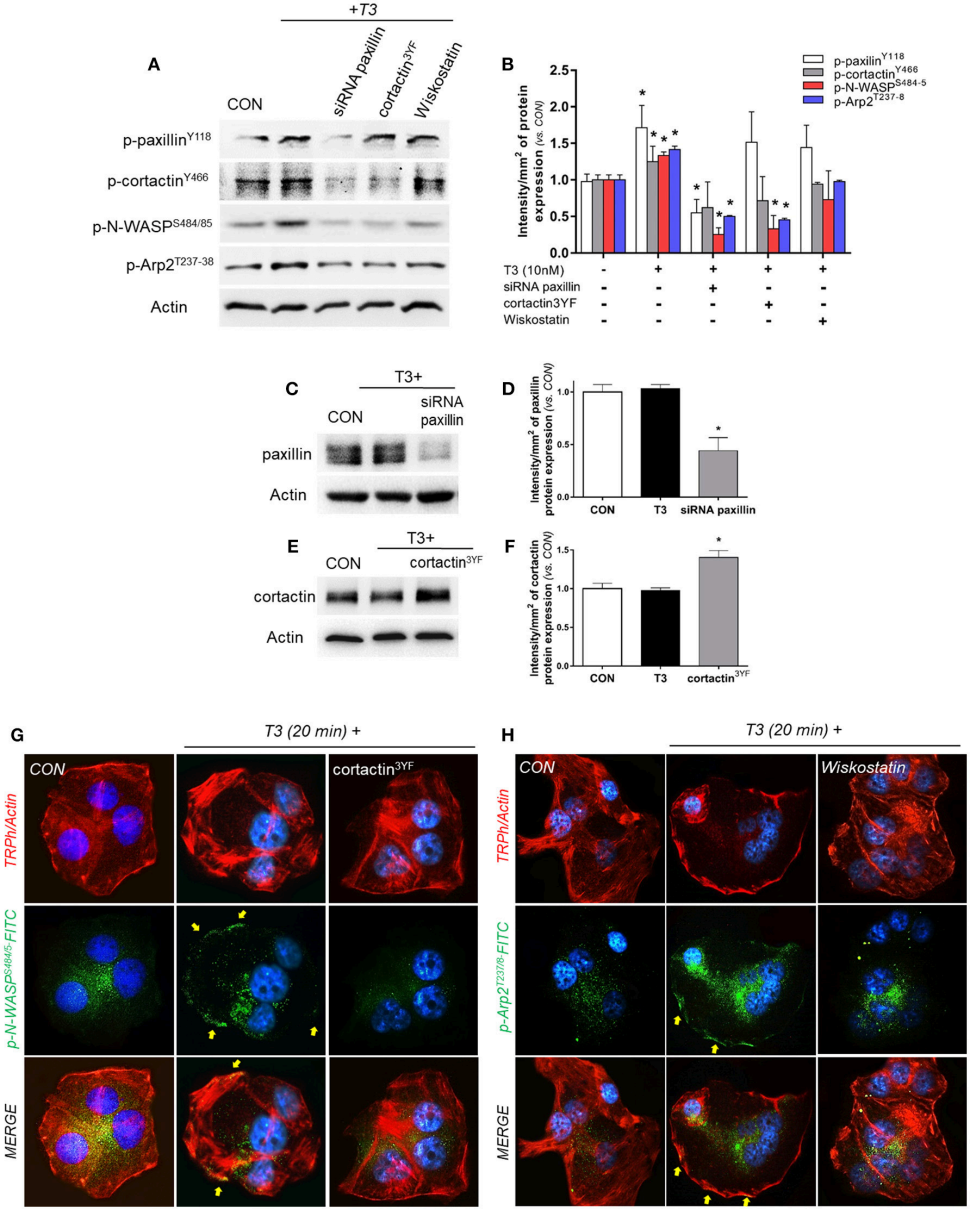
T3 signals to paxillin, cortactin, N-WASP and Arp2/3 complex. **(A,B)** T-47D cells were incubated in the presence of 10 nM T3 for 20 min with or without silencing of paxillin with specific siRNAs and/or inhibition of cortactin and N-WASP. Actin, phospho-paxillin^Y118^, p-cortactin^Y466^, p-N-WASP^S484/485^ and p-Arp2^Y237^ were assayed in cell extracts. The densitometry values were adjusted to actin intensity, then normalized to the control sample. **(C–F)** T-47D cells were transfected, with paxillin-targeted siRNAs or the dominant negative constructs of cortactin (*cortactin3YF)* and incubated with T3 (10 nM) for 20 min. Paxillin protein expression was detected by Western blot, and actin intensity was used as loading control. Paxillin and cortactin densitometry values were adjusted to actin intensity, then normalized to the control sample. *P < 0.05 vs. corresponding control. **(G)** Cells were stained with anti-phospho-N-WASP^S484/485^ linked to FITC (green) and **(H)** phospho-Arp2^T237/8^ linked to FITC (green), filamentous actin was stained with phalloidin linked to Texas Red and nuclei were counterstained with DAPI. CON, Control. Yellow arrows indicate membrane-localized p-N-WASP^S484/485^ and p-Arp2^T237/8^. All experiments were performed in triplicate with consistent results; representative images are shown.

In order to continue elucidating the signaling by which T3 enhances BC cell movement, we evaluated the role of paxillin toward three fundamental components of actin nucleation: cortactin, N-WASP and Arp2/3 complex. We observed that the rapid treatment with 10 nM of T3 (20 min) significantly increased the phosphorylation levels of paxillin, cortactin, N-WASP and the subunit Arp2 (Arp2/3 complex) (Figures 4A,B). Paxillin phosphorylation was only prevented by the use of the specific siRNA vs. paxillin, whereas phospho-cortactin was inhibited by the siRNA vs. paxillin and its dominant-negative cortactin^3YF^ construct (cortactin^3YF^), but not with the specific inhibitor of N-WASP, Wiskostatin. Finally, N-WASP and Arp2 phosphorylation were markedly reduced by the silencing of paxillin, blockade of cortactin^3YF^ and the inhibition of N-WASP (Figures 4A,B), suggesting that integrin αvβ3 signals to Arp2/3 complex via FAK, paxillin, cortactin, and N-WASP.

The efficacy of transfections was assayed by Western blot. The siRNA of paxillin significantly reduced its expression (Figures 4C,D), whereas the expression of cortactin^3YF^ construct significantly increased the amount of cortactin present in these BC cells (Figures 4E,F).

We next evaluated the subcellular localization of phosphorylated N-WASP^S484/485^ and Arp2^T237/238^. In control cells, phospho-N-WASP and phospho-Arp2 were weakly distributed throughout the cytoplasm (Figures 4G,H). T3 exposure for 20 min increased N-WASP^S484/485^ and Arp2^T237/238^ phosphorylation and translocation to the plasmatic membrane (Figures 4G,H). This relocalization was prevented by the use of cortactin3YF (Figure 4G) and Wiskostatin (Figure 4H).

### T3 Enhances Cell Adhesion and Invasion via Paxillin/Cortactin/N-WASP/Arp2/3 Complex

**Figure 5:**
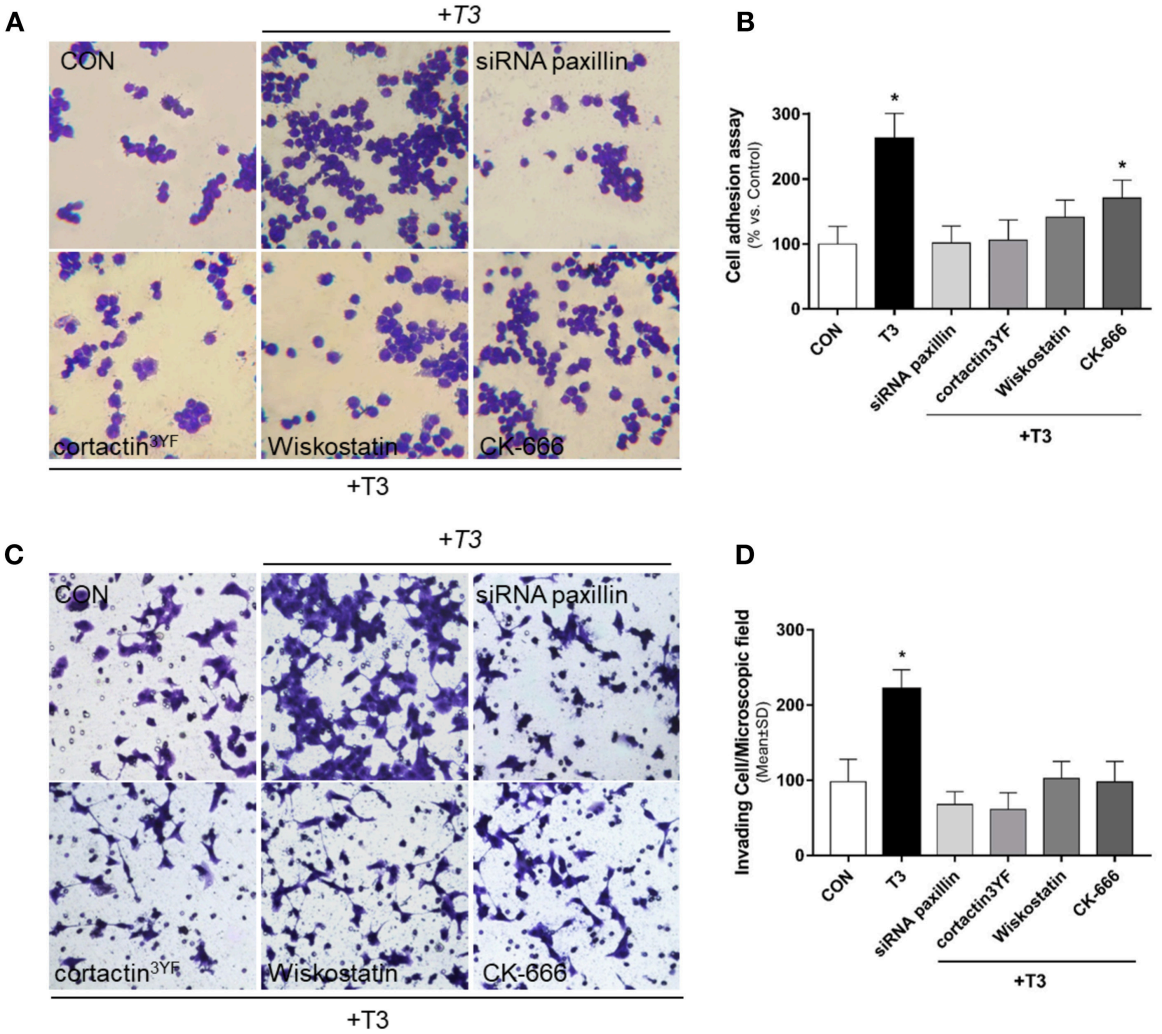
BC cell adhesion and invasion is triggered by T3 via paxillin/cortactin-N-WASP/ Arp 2/3 Complex pathway. T-47D cells were treated with T3 (10 nM) for 2 h **(A)** and 48 h **(C)** in the presence or absence of Wiskostatin or CK-666, and transfected with siRNAs vs. paxillin or mutant constructs for cortactin (*cortactin3YF*). **(A)** After the treatment, cells were placed on coverslips previously covered with gelatin and a cell adhesion assay was performed. Representative images of adhered cells are shown. **(B)** Percentage of attached cells vs. CON, Control cells. Experiments were performed in triplicate; *P < 0.05 vs. CON. **(C)** Breast cancer cell invasion through matrigel was assayed with invasion chambers. Representative images in chambers with matrigel are shown. **(D)** Invading cells were counted in the central field of triplicate membranes. *P < 0.05 vs. CON.

We performed an adhesion and three-dimensional invasion assay using Matrigel to determine the ability of cancer cells to adhere and invade the surrounding environment. Treatment with T3 (10 nM) enhanced the capacity of BC cells to adhere (Figures 5A,B, yellow arrows) and invade ECM (Figures 5C,D, yellow arrows). This ability was drastically diminished in treatments where specific inhibitors were used, such as siRNA paxillin, cortactin^3YF^, Wiskostatin and the specific inhibitor of the Arp2/3 complex (CK-666) (Figures 5A,D). These results support the concept that paxillin, cortactin, N-WASP and Arp2/3 Complex are involved in adhesion and cellular invasion processes triggered by T3.

## Discussion

In the last years, several studies have evidenced a link between thyroid hormones (TH) and cancer development (21). Because TH regulate growth, differentiation, development and metabolism, altered levels of these hormones could play a significant role in the development and progression of diverse types of cancer, including breast cancer (BC). Published works about the relationship between hyper- and hypothyroidism and the incidence of BC are controversial. Some authors consider hypothyroidism an important enhancer factor in invasion and metastasis (22), other studies suggest that hypothyroidism does not affect, or, rather, reduces the risk of developing the disease (23), whereas other not found association between hypothyroidism or hyperthyroidism and BC (24). In addition, an important association between hyperthyroidism and the risk of developing BC has been established (25, 26). Søgaard et al. (27) described an increased risk of BC in women with hyperthyroidism and a slightly reduced risk in women with hypothyroidism, suggesting that a correlation exists between TH levels and BC risk.

Furthermore, a significant positive associations between higher prediagnostic T3 levels, larger tumors and occurrence of lymph node metastases have been demonstrated, suggesting that this association, can be related to both a higher incidence and more aggressive forms of BC, increasing BC mortality (28).

For this reason, in this work we evaluated the effect of T3 in a supraphysiological concentration (10 nM) taking into account that altered levels of this hormone could be related with BC.

We have previously shown that reducing T3 levels downregulates the expression of key cell motility regulators, such as Src, FAK, and PI3K kinase, in T-47D BC cells (11). In the present work we observed that treatment with T3 (10 nM) results in a maximal phosphorylation of cortactin and N-WASP (Figures 2B,C), which suggests that altered levels of T3 affect BC cells' ability to induce the adhesion, migration and invasion processes.

As a first approach, we studied the involvement of T3 in the epithelial-mesenchymal transition (EMT), a key step for the early development of metastasis. We observed a progressive reduction of E-cadherin expression and an increase in vimentin expression in a time-dependent manner, being maximal at 1 h and returning to basal levels at 12 and 24 h. We also showed, by means of immunofluorescence experiments, that these changes were accompanied by a reduction of E-cadherin at the plasma membrane and an increase in the intensity of vimentin filaments in the cytoplasm. Lamouille et al. (5) have described that during the EMT initiation, E-cadherin expression is reduced because it is cleaved at the plasma membrane and degraded in the cytoplasm. This could explain the protein level reduction observed in this work. Furthermore, an increased expression of intermediate filaments, such as vimentin, is necessary to determine the beginning of the transition (29), by promoting the directional cell migration by regulating microtubule polarity and focal adhesion dynamics (30). Previous works have also reported this altered expression of E-cadherin/vimentin in response to TH. Weingarten et al. (6) have determined that 1-4 h of T3 and T4 treatment drastically increases the expression of vimentin and reduces E-cadherin mRNA level to half of its basal level in OVCAR-3 and SKOV-3 ovarian cancer cell lines. They demonstrated that the modifications in EMT proteins were mediated by integrin αvβ3 membrane receptor, which is consistent with our results (6). The EMT is an orchestrated sequential steps process in which cell-cell and cell-extracellular matrix (ECM) interactions are modified to release epithelial cells from the surrounding tissue, along with actin cytoskeleton rearrangements to confer cells the ability to migrate through a three-dimensional ECM. In fact, TH are capable of modify the expression of several and crucial components of ECM. In mouse mammary epithelial cells, T3 increases the expression of the proteases stromelysin 1 and 2 and stimulates their activity leading to a gelatinolytic activity of type IV collagenase (31). In human hepatoma cells and fibroblast, T3 induces fibronectin expression by activating hypoxia-inducible factor-1 (HIF-1) (32). Furthermore, in astrocytes TH regulates integrin interactions with ECM proteins like laminin, being essential for their migration during brain development (33, 34). These studies indicate that TH may exert an integrated regulation of tumor progression by modifying the ECM, the EMT and the signaling pathways implicated in cell migration and invasion. Our study, specifically reveals that T3 enhance the EMT and this leads to an important cell cytoskeleton reorganization, increasing cellular motility and enabling cells to develop an invasive phenotype driven by T3.

Another finding of this work is that T3 stimulates morphological changes that depend on the generation of dynamic structural modifications of the actin cytoskeleton reorganization, via actin polymerization/depolymerization, in association with specialized membrane structures, such as lamellipodia, filopodia and membrane ruffles. These structures are fundamental to promote cell adhesion, migration and invasion processes.

Cell adhesion is carried out by different integrins including αvβ3 that recruits Src and FAK kinases, which are critical for the formation of focal adhesion (FAs) complexes (16, 34, 35). Cohen et al. (36) have shown that T3 and T4 regulate adhesion, migration and matrix metalloproteinase activity via integrin α_v_/β_3_ in myeloma cells. We have previously reported that T3, via integrin αvβ3, represents the starting platform to activate Src, FAK, and PI3K, which leads to increased BC cell motility (11). We have not evaluated the use of Tetrac to block cell adhesion, migration and invasion, but Weingarten et al. (6) are currently developing a nano-particle antagonist of thyroid-integrin binding that could represent a novel agent that may limit the metastasic potential of cancer cells. For this reason, we continued evaluating the non-genomic effects of T3 on several kinases and scaffold proteins related to cell movement, such as paxillin. Paxillin has many binding partners and acts as a pivot molecule between the formation of focal adhesion complex and the actin nucleation, key steps in the regulation of cell motility (3, 37). Our results show that T3 induces paxillin phosphorylation whereas the specific integrin αvβ3 receptor antagonist Tetrac inhibits this action. Once paxillin is activated by T3, it translocates to sites where FAs are formed. This effect is dependent on integrin αvβ3, Src and FAK proteins. This finding is in agreement with our previous work, in which we have shown that paxillin is recruited to FAs by Src and FAK in response to rapid treatments of BC cells with estradiol (37). Similarly, Deramaudt et al. (38) have shown that a FAK mutant construct deficient in binding paxillin disrupts FA formation and drastically reduces cell adhesion, migration and invasion of mouse fibroblasts. When paxillin is phosphorylated and recruited to FAs, it becomes a docking site for many downstream signaling molecules, among them the actin nucleation regulators cortactin and N-WASP (37, 39).

Actin nucleation is crucial for directional cell motility through the actin polymerization process. The latter involves the generation and turnover of actin filaments, which form subcellular structures (lamellipodia and filopodia) that are key for cell movement. In this context, many signaling pathways drive actin nucleation by regulating actin-binding protein activity. In order to be functional, Arp2/3 complex needs to be activated by proteins called nucleation promoting factors or NPFs. In this work we evaluated the role of T3 on two main NPFs, cortactin and N-WASP. We identified the recruitment of the FAK/paxillin/cortactin by T3, which is a step required for N-WASP and Arp2/3 complex phosphorylation and translocation to the plasma membrane. Blocking this event by using specific inhibitors or mutant constructs drastically affects cell adhesion and invasion, which highlights the importance of actin nucleation proteins in tumor progression. These findings emphasize the relevance of the cortactin/N-WASP/Arp2/3 complex phosphorylation and regulation for cancer metastasis. The discovery that cortactin/N-WASP controls the Arp2/3 complex via thyroid hormones may thus offer novel insights to better understand the action of these hormones on BC metastasis, although there is considerable evidence of the role of TH in enhancing metastasis in several types of cancer (40). The use of specific NPF inhibitors could thus be an attractive alternative to counteract the ability of BC cells to metastasize. Although no drug is available to target cortactin, Dasatinib is currently used to disrupt the Src/cortactin signaling pathway for blocking BC metastasis (41). A recent study demonstrated that using nanobodies to target the VCA domain of N-WASP results in a diminished invasiveness of breast, prostate, and head and neck squamous cancer cells by disrupting N-WASP/Arp2/3 complex interaction (42).

Additionally, we also studied the phosphorylation of Arp2 subunit, which is fundamental to the fully functioning of Arp2/3 complex (43). Our results suggest that paxillin, cortactin, and N-WASP relay signals from integrin αvβ3 to the Arp2/3 complex.

One limitation of this study is the use of only one BC cell line. Also, it would be interesting to validate these findings in other models, mainly in primary cell cultures because integrin α_v_/β_3_ is universally expressed in cancer cells.

**Figure 6:**
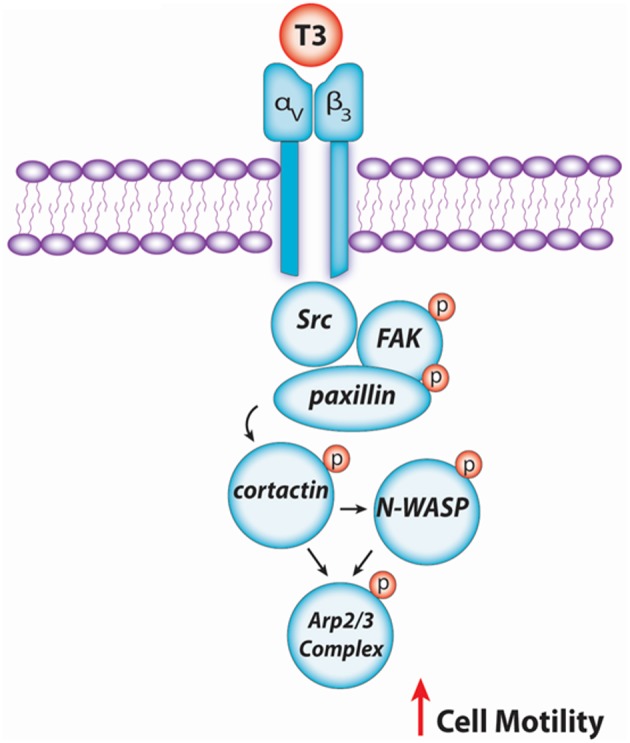
Schematic signaling cascade triggered by T3 promoting BC cell migration and invasion. Binding of T3 to the specific membrane integrin αvβ3 receptor induces phosphorylation of the Src/FAK/paxillin/cortactin/N-WASP/Arp2/3 Complex cascade, promoting BC cell motility.

In conclusion, our findings provide new insights about the non-genomic action of T3 in BC cell progression via integrin αvβ3/FAK/paxillin/cortactin/N-WASP/Arp2/3 complex (Figure 6). We observed how T3 induces rapid alterations in the plasma membrane, leading to a rearrangement of the actin cytoskeleton and consequent formation of structures related with cell motility, increasing cell adhesion, migration, and invasion in T-47D BC cells. These findings could be helpful to develop new drugs that interfere with the ability of breast tumors to diffuse locally or at distant sites in patients with thyroid disorders and breast cancer, counteracting BC cell progression by controlling circulating T3 levels.

## Author Contributions

IU carried out different experiments, cell culture and treatments. JC performed immunofluorescence and migration assays. MF was instrumental in funding the study and participated to the writing of the manuscript. AS planned and funded the project, supervised the experiments, wrote the paper.

### Conflict of Interest Statement

The authors declare that the research was conducted in the absence of any commercial or financial relationships that could be construed as a potential conflict of interest.
